# Tumour necrosis factor α (TNF-α), interleukin-6 (IL-6) and their soluble receptors (sTNF-α-Rp55 and slL-6R) serum levels in systemic lupus erythematodes

**DOI:** 10.1155/S0962935196000609

**Published:** 1996-12

**Authors:** E. Robak, A. Sysa-Jędrzejowska, T. Robak, H. Stępień, A. Woźniacka, E. Waszczykowsk

**Affiliations:** 1 Department of Dermatology and Venerology Medical University of Łódź Krzemieniecka 6 Łódź 94-017 Poland; 2 Department of Hematology Medical University of Łódź Pabianicka 62 Łódź 93-513 Poland; 3 Department of Experimental Endocrinology Medical University of Łódź Sterlinga 5 Łódź 91-425 Poland

## Abstract

We investigated a possible association between serum concentrations of tumour necrosis factor α (TNF-α), interleukin-6 (IL-6) and their soluble receptors (sTNF-α-Rp55 and sIL-6R) using an enzyme-linked immunosorbent assay (ELISA) in 55 patients with systemic lupus erythematodes (SLE) and 16 healthy controls. We also examined a possible association between the serum levels of these peptides and SLE activity, as well as TNF-α and IL-6 concentrations and the levels of their soluble receptors. The median concentrations of TNF-α, sTNF-α-Rp55 and IL-6 were significantly higher in SLE patients than in normal individuals. In contrast, there was no difference between the serum level of sIL-6R in both groups. We found positive correlations between the serum concentrations of TNF-α and IL-6 as well as their soluble receptors and disease activity. There were also correlations between TNF-α and sTNF-α-Rp55 as well as IL-6 and sIL-6R serum levels in SLE patients but there were no such correlations in the normal control group. In conclusion, an increase in the serum levels of TNF-α, sTNF-α-Rp55 and IL-6 may become useful markers for SLE activity. Patients with SLE have sIL-6R serum concentration similar to that as in normal individuals. However, it correlates with disease activity and the level of IL-6.


Mediators of Inflammation, 5, 435–441 (1996)

### Tumour necrosis factor α (TNF-α), interleukin-6 (IL-6) and their soluble receptors (sTNF-α-Rp55 and sIL-6R) serum levels in systemic lupus erythematodes

E. Robak,[1] A. Sysa-Jędrzejowska,[1] T. Robak,[2,CA] H. Stępień,[3] A. Woźniacka[1] and E. Waszczykowska[1]

[1]Department of Dermatology and Venerology, Medical University of Łódź, Krzemieniecka 6, 94-017 Łódź; [2]Department of Hematology, Medical University of Łódź, Pabianicka 62, 93-513 Łódź; [3]Department of Experimental Endocrinology, Medical University of Łódź, Sterlinga 5, 91-425 Łódź, Poland

[CA]Corresponding Author
Tel/Fax: (+48) 42 84 6890

**We investigated a possible association between serum concentrations of tumour necrosis factor α (TNF-α), interleukin-6 (IL-6) and their soluble receptors (sTNF-α-Rp55 and sIL-6R) using an enzyme-linked immunosorbent assay (ELISA) in 55 patients with systemic lupus erythematodes (SLE) and 16 healthy controls. We also examined a possible association between the serum levels of these peptides and SLE activity, as well as TNF-α and IL-6 concentrations and the levels of their soluble receptors. The median concentrations of TNF-α, sTNF-α-Rp55 and IL-6 were significantly higher in SLE patients than in normal individuals. In contrast, there was no difference between the serum level of sIL-6R in both groups. We found positive correlations between the serum concentrations of TNF-α and IL-6 as well as their soluble receptors and disease activity. There were also correlations between TNF-α and sTNF-α-Rp55 as well as IL-6 and sIL-6R serum levels in SLE patients but there were no such correlations in the normal control group. In conclusion, an increase in the serum levels of TNF-α, sTNF-α-Rp55 and IL-6 may become useful markers for SLE activity. Patients with SLE have sIL-6R serum concentration similar to that as in normal individuals. However, it correlates with disease activity and the level of IL-6.**

**Key words:** Disease activity, Interleukin-6, Soluble interleukin-6 receptor, Soluble tumour necrosis factor α receptor p55, Systemic lupus erythematodes, Tumour necrosis factor α

### Introduction

Systemic lupus erythematodes (SLE) is a disorder of generalized autoimmunity with multisystem organ involvement and autoantibodies against nuclear, cytoplasmic and cell surface antigens. The disease is characterized by B cell activation and autoantibody formation.[1,2] However, increasing evidence indicates to a critical role of T cells, particularly $CD4^+$ cells, in inducing B cell hyperactivity.[3] To explain the mechanisms responsible for immune dysregulation in SLE, cytokines have become the object of intensive studies.[2–4]

Cytokines are a group of polypeptides synthesized by the host in response to different injuries. They may affect different cell functions and are involved in immunity and inflammatory response. Among the many cytokines, interleukin-1 (IL-1), interleukin-6 (IL-6), interferon-γ (IFN-γ) and tumour necrosis factor-α (TNF-α) seem to play the most important role in the pathogenesis of inflammatory and autoimmune diseases.[4–6]

The intracellular signals for the response to cytokines are provided by cell surface receptors.[7,8] Cytokine receptors exist also in soluble forms, apparently deriving from proteolytic cleavage from the cell surface forms.[9]

Several preliminary studies show conflicting data on the correlations between some inflammatory cytokines and their soluble receptors' levels in the serum of patients with SLE and disease activity. An initial report suggested that TNF-α was not elevated in the serum of lupus patients except during infection.[10] Subsequently, elevated TNF-α levels in SLE patients compared with controls[11] and its correlation with disease activity was reported.[12] Similarly, there have been several reports suggesting that the levels of IL-6 are elevated in active lupus.[13–15] However, other reports do not support these observations.[16]

The biological role of soluble cytokine recep-

© 1996 Rapid Science Publishers tors is not fully elucidated. They may modify ligand concentrations by serving as stabilizing binding proteins, may downregulate membrane receptor number as a mechanism of genesis, and may specifically inhibit ligand-receptor association in the extracellular space.[16]

Two receptors of different molecular structure have been recognized for TNF-α: type I or TNF Rp55, of 55–60 kDa and type II or TNF-Rp75, with 75–80 kDa molecular mass.[7] Soluble forms of these two TNF receptors (sTNF-Rs) have been identified in serum and urine.[9,17]

Neutrophils, activated T-cells and monocytes are the main sources of sTNF-α-Rs.[17] Their concentration in serum increases significantly in inflammatory and cancer diseases.[17,18] Both types of sTNF-αRs have been reported to be elevated in patients with SLE.[19–21] The sTNF-α-Rs levels correlate with disease activity and the impairment of renal function may contribute to a rise of their serum concentrations.[19] These sTNF-α-Rs appear to be biologically functional because they inhibited TNF-α action in cytotoxicity assays.[21]

The cellular IL-6R complex consists of two different proteins, an 80-kDa ligand binding glycoprotein (IL-6R) and a 130-kDa protein (gp 130) involved in cellular signal transduction.[22] Müllberg *et al.* have recently shown that two subunits of the IL-6R complex are proteolytically cleaved and released from the cell as soluble receptor proteins.[23,24] Their concentrations seem to be elevated during infections, inflammatory and neoplastic diseases. In contrast to other soluble cytokine receptors, the sIL-6R together with IL-6 acts agonistically on cells that express gp 130. To our best knowledge, the serum level of sIL-6R in SLE patients has not been investigated so far.

In the present study we measured the serum concentrations of TNF-α, and IL-6 and their soluble receptors (sTNF-α-R55 and sIL-6R) in 55 patients with SLE using ELISA assay. We also correlated the serum levels of these proteins with disease activity.

### Patients and Methods

### Patients

A total of 55 unselected patients with SLE, 53 women and two men all fulfilling the 1982 revised criteria defined by the American Rheumatism Association (ARA)[25] were included in our study. Their mean age was 40.5 years (range 20–66 years). The mean duration of their disease was 87.4 months (range 4 months to 30 years). Four patients were never treated with immunosuppressive agents. Fifty-one patients were treated with steroids and two of them with azatioprine for some time during the course of their disease, but 20 of them had not been treated for at least 4 weeks before the investigation of cytokines.

We included in the study patients with active and inactive disease. Disease activity was scored during the visit to the outpatient clinic according to the method proposed by Liang *et al.* in our modification.[26] Each patient was assessed on two separate occasions 2–4 weeks apart. The system Systemic Lupus Activity Measure (SLAM) includes 24 clinical manifestations and eight laboratory parameters.

Parameters of immune function were not included. Maximal score points in this system is 84. We assumed the score of 0–10 points for inactive disease, and a score of over 10 points for active disease. Our group of patients included 13 inactive patients and 42 active disease patients (Table 1).

The control group of 15 healthy volunteers was also studied. They were 14 women and one man, aged from 25 to 50 years (median 39 years).

### Laboratory tests

On the day of blood sampling for TNF-α, IL-6 and their soluble receptors the following laboratory parameters were analysed: complete blood cell count (CBC), erythrocyte sedimentation rate (ESR), blood urea nitrogen and creatinine levels, fibrinogen level, partial thromboplastin

**Table 1.** Clinical characteristics of patients with systemic lupus erythematodes (symptoms according to Liang *et al.*[27])

| Symptom | No. of patients | (%) |
|---|---|---|
| Total | 55 | (100) |
| Active | 42 | (76.4) |
| Inactive | 13 | (23.6) |
| Fever | 7 | (12.7) |
| Skin symptoms | 49 | (89.1) |
| Reticuloendothelial system involvement | 27 | (49.1) |
| Pulmonary symptoms | 7 | (12.7) |
| Cardiovascular symptoms | 96 | (83.6) |
| Neurologic disorder | 44 | (80.0) |
| Arthritis | 43 | (78.2) |
| Renal disorder (creatinine > 1.3 mg/dl) | 7 | (18.2) |
| Antinuclear antibodies | 47 | (85.4) |
| Anaemia (Hb < 12 g/dl) | 17 | (30.9) |
| Leukopenia (< $3.5 \times 10^9$/l WBC) | 16 | (29.1) |
| Thrombocytopenia (< $150 \times 10^9$/l platelet) | 18 | (32.7) |
| Raised ESR (> 25 mm/h) | 37 | (67.3) |
| Fibrinogen (> 400 mg/dl) | 18 | (32.7) |
| Treatment with steroids during the study | 35 | (63.6) |

time (PTT), immunoglobulins (IgG, IgA, IgM) and complement ($C_3$, $C_4$), urine levels and the level of anti-DNA antibodies.

### Serum sampling

Venous blood samples for TNF-α and TNF receptor determinations were collected at the time of clinical assessment into pyrogen-free tubes, and centrifuged within 20 min after being allowed to clot at −4°C for 1 h at 2000 *g* for 10 min. The serum obtained was divided into aliquots and stored at −25°C until assayed. TNF-α, IL-6 and sTNF-α-Rp55 sIL-6R were assayed by specific commercially available, enzyme-linked (ELISA) assay kits (Quantikine, R&D Systems Inc., USA) in accordance with the manufacturer's protocols.

In each assay, the appropriate recombinant human cytokine was used to generate the standard curve. Sensitivity of the assay for TNF-α was 0.5 pg/ml; for sTNF-α-Rp55, 7.8 pg/ml; for IL-6, 0.3 pg/ml. Serum for IL-6R concentration measurement was diluted 40 times and its level was measured between 7.8 and 500 pg/ml. The concentrations of cytokines and soluble receptors in the samples were determined by interpolation from the standard curve.

### Statistical analysis

Differences in parameters between groups were evaluated with Student's *t*-test. The chi-squared and Fisher's exact tests were used to analyse the relationship between cytokines and soluble receptors serum values and SLE activity.

Internal distribution data were analysed by Student's *t*-test. Correlations were evaluated using the Spearman rank-sum correlation coefficient and linear regression calculated with the least-squares method.

Results are presented with $R^2$ coefficients. Comparisons and correlations were considered significant when $p < 0.05$.

### Results

Table 2 shows the results of measurement of TNF-α, sTNF-α-Rp55, IL-6 and sIL-6R in the serum of 55 patients with SLE and 16 normal individuals. In the group of SLE patients 42 were with active and 13 with inactive disease according to Liang *et al.*'s scoring system with our modification. The highest TNF-α concentration was in active SLE patients (median 5.1 pg/ml) and the lowest in the healthy control group (0.6 pg/ml) ($p < 0.001$). However, a significant difference in the serum TNF-α level was also noted between active and inactive SLE patients ($p < 0.02$). The sTNF-α-Rp55 level was also higher in SLE patients than in normal persons (mean values 1695.4 and 749.4, respectively); however, there was no significant difference between active and inactive SLE.

We found a positive correlation between TNF-α concentration and SLE activity score ($R^2 = 0.3512$, $p < 0.05$) and sTNF-α-Rp55 level and disease activity ($R^2 = 0.2559$, $p < 0.05$) (Fig. 1). There was also a positive correlation between TNF-α and sTNF-α-Rp55 concentrations in the serum of SLE patients (Fig. 2) and a lack of such correlations in normal controls (data not shown).

The serum level of IL-6 was higher in SLE

**Table 2.** Serum levels of TNF-α, sTNF-α-Rp55, IL-6 and sIL-6R in patients with SLE and normal controls. Mean values in pg/ml ± SD and range in parentheses

| Group Cytokines and receptors | All SLE patients $n = 55$ a | Active SLE $n = 42$ b | Inactive SLE $n = 13$ c | Normal control $n = 16$ d | Statistical analysis |
|---|---|---|---|---|---|
| TNF-α | 4.5 ± 7.1 | 5.1 ± 7.8 | 2.7 ± 3.3 | 0.6 ± 0.4 | a & d $p < 0.001$* |
| | | | | | b & d $p < 0.01$* |
| | (0.4–54.0) | (0.6 ± 54.0) | (0.4–12.8) | (0.1–1.6) | c & d $p < 0.02$* |
| | | | | | b & c $p < 0.02$* |
| sTNF-α-Rp55 | 1695.4 ± 1287.4 | 1843.2 ± 1412.3 | 1217.8 ± 518.4 | 749.4 ± 129.3 | a & d $p < 0.001$* |
| | | | | | b & d $p < 0.001$* |
| | (697.0–9458.1) | (775.1–9458.1) | (697.0–2699.1) | (487.0–1002.0) | c & d $p < 0.01$* |
| | | | | | b & c $p > 0.05$ |
| IL-6 | 15.4 ± 48.1 | 19.7 ± 54.3 | 1.7 ± 1.8 | 1.3 ± 1.1 | a & d $p < 0.01$* |
| | | | | | b & d $p < 0.01$* |
| | (0.1–312.8) | (0.6–312.8) | (0.1–6.1) | (0.3–4.8) | c & d $p > 0.05$ |
| | | | | | b & c $p < 0.02$* |
| sIL-6R | 972.8 ± 326.9 | 1000.5 ± 351.9 | 883.2 ± 203.2 | 919.9 ± 186.2 | a & d $p > 0.05$ |
| | | | | | b & d $p > 0.05$ |
| | (114.6–2266.2) | (114.6–226.2) | (524.6–1293.0) | (610.6–1305.2) | c & d $p > 0.05$ |
| | | | | | b & c $p > 0.05$ |

*Differences statistically significant.

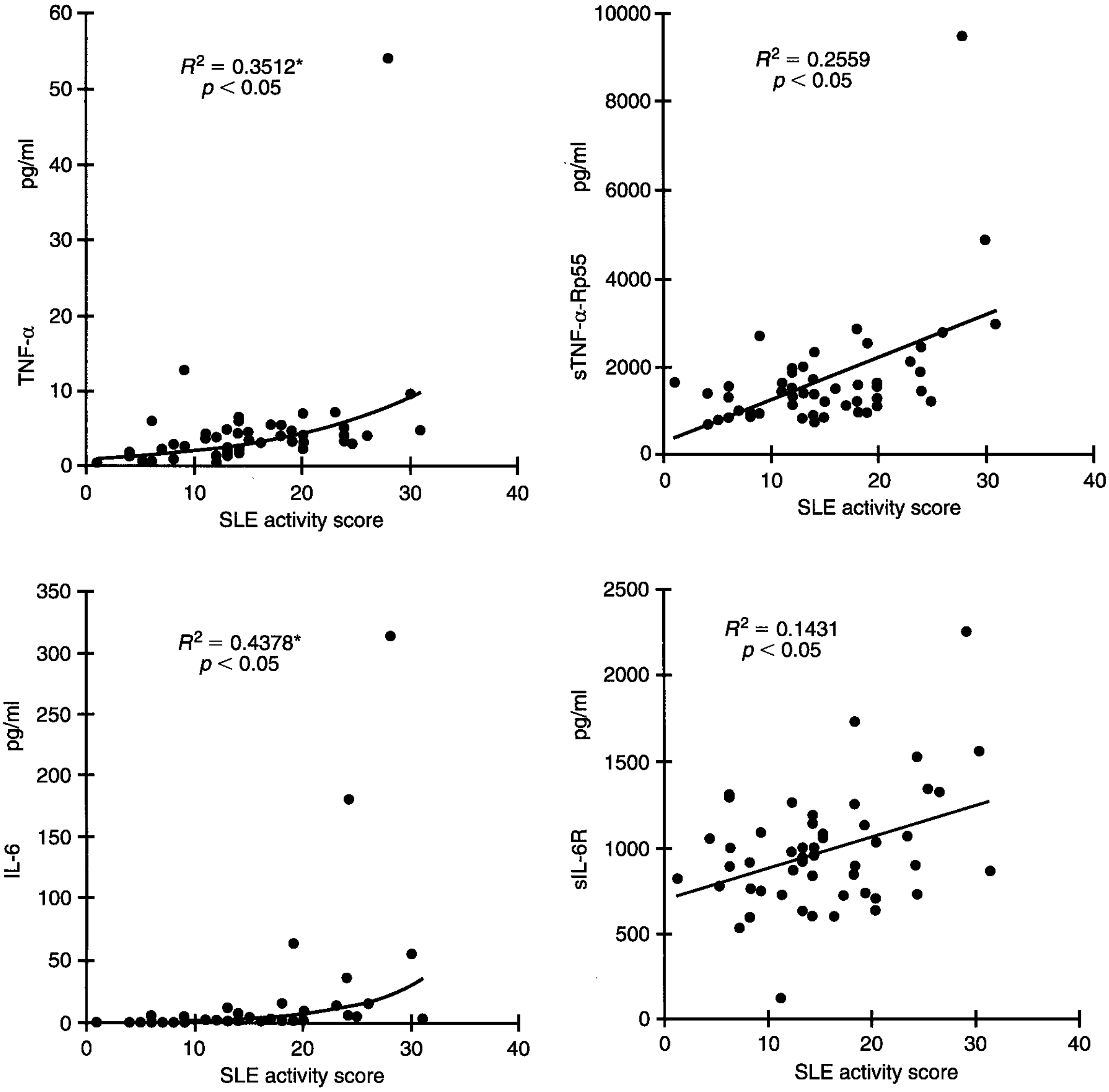

FIG. 1. Correlations between serum TNF-α sTNF-α-Rp55, IL-6 and sIL-6R with SLE activity. *Exponential regression.

patients (mean 15.4 pg/ml) than in the control group (mean 1.3) ($p < 0.01$). However, we found no difference in serum IL-6 concentrations in inactive SLE compared with normal individuals (mean 0.8 and 1.0 respectively, $p > 0.05$). The median serum levels of sIL-6R were similar in SLE patients and in the control group as well as in active and inactive SLE ($p > 0.05$) (see Table 2). However, there was a positive correlation between IL-6 and sIL-6R concentrations and SLE activity (Figs 1 and 2). We also found a significant positive correlation between the levels of IL-6 and sIL-6R in SLE patients ($R^2 = 0.1954$, $p < 0.001$) and lack of such correlations in the normal group (data not shown).

We also analysed the relationship between serum concentrations of TNF-α and IL-6 as well as sTNF-α-Rp55 and sIL-6R (Fig. 2). We observed positive correlations between their parameters ($R^2 = 0.7008$, $p < 0.001$ and $R^2 = 0.3683$, $p < 0.001$, respectively).

### Discussion

The objective of our study was to evaluate the concentrations of two cytokines, TNF-α and IL-6, whose role is inflammatory and immunologic processes is fairly well known, as well as their soluble receptors, sTNF-α-Rp55 and IL-6R, in patients with SLE. In the study, the correlation between concentrations of these peptides and the activity of SLE as well as the incidence rate of particular clinical and laboratory symptoms of the disease was analysed. We have demonstrated that the concentration of both TNF-α and IL-6 in the serum of SLE patients is higher than in healthy individuals, and that it displays a

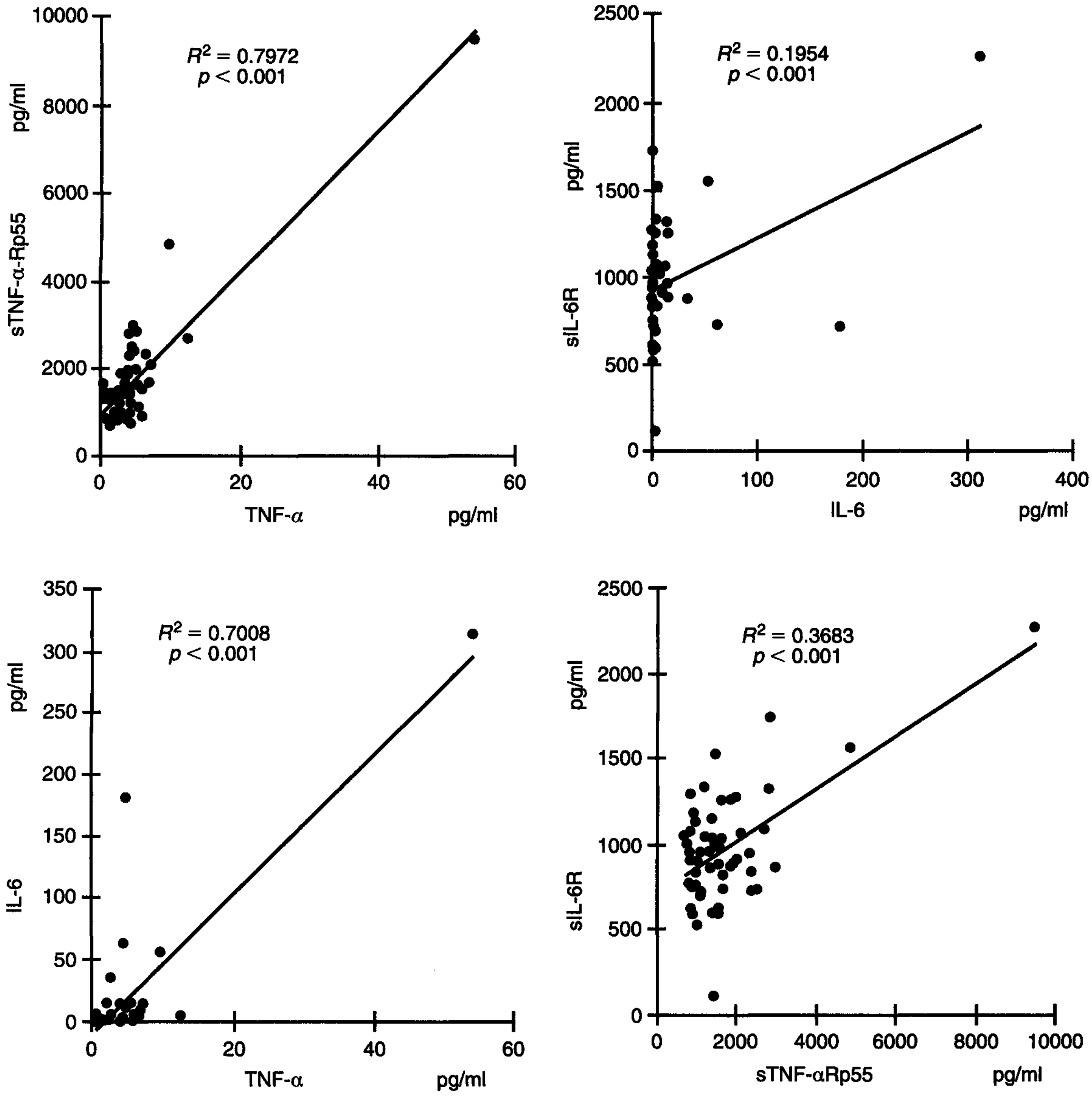

FIG. 2. Correlations between TNF-α and sTNF-α-Rp55, IL-6 and sIL-6R, TNF-α and IL-6 sTNF-α-Rp55 and sIL-6R serum concentrations in patients with SLE.

positive correlation with the disease activity. These results are consistent with the observations of other authors.[11–15] However, in contrast with our studies, Gordon and Emery did not observe any correlation between TNF-α concentration and SLE activity,[5] and Metsariune *et al.* did not find IL-6 concentrations to be higher in SLE patients than in healthy controls.[16] The above differences may arise due to a different sensitivity and specificity of the methods applied to assay cytokines (ELISA and bioassay), and to the number of patients under investigation.

It seems justified to assume that both TNF-α and IL-6 play a significant role in the pathogenesis of SLE. *In vitro* studies show a disturbed production of TNF-α in SLE. Malave *et al.* demonstrated a decrease in its production by lectin-stimulated mononuclear cells in SLE patients despite the fact that in these cells the mRNA is higher for TNF-α than in healthy individuals.[27] Moreover, monocytes in SLE patients can spontaneously secrete TNF-α in *in vitro* culture, with immunologic complexes being a strong stimulator of its production by these cells.[28] In SLE, a higher TNF-α concentration was also found in the disease affected tissues compared with healthy tissues. It should also be added that excessive TNF-α production may be conditioned genetically. It may be associated with the presence of TNF-α gene, and this presence can in consequence condition the development of autoimmune diseases, including SLE. The above observations may account for a higher TNF-α concentration in the serum of patients with an active form of SLE, as well as point to a significant role of this cytokine in the pathogenesis of the disease.

In SLE, anomalies were also observed in the production process of IL-6 and the response of target cells to its activity. Lymphocytes T and B, as well as monocytes of SLE patients produce IL-6 in greater quantities than their equivalents in healthy individuals; this intensification in the production may be induced by immunologic complexes.[13] Another observation was that lymphocytes B obtained from SLE patients were more sensitive to the effect of IL-6 in comparison with lymphocytes from healthy controls; thus, this cytokine may be for them an autocrinic growth factor.[29] Of significance may be the observation that in SLE patients there is no evidence of subpopulation of cells specifically inhibiting IL-6 expression, and that the majority of lymphocytes B exhibit spontaneous expression of the receptors for this cytokine.[30]

In our studies, the sTNF-α-Rp55 concentration in serum was higher in SLE patients than in healthy controls and correlated positively with the activity of the disease, which is in agreement with observations by other authors.[19–21,31] However, we did not observe differences in the sIL-6R concentration between SLE patients and healthy controls, although the concentration of this cytokine corrrelated positively with the disease activity.

The studies conducted demonstrate that sTNF-α-Rp55, along with TNF-α and IL-6, can be regarded as a marker of SLE activity, whereas sIL-6 cannot serve as such. Another marker of the disease activity is soluble receptor IL-2 (sIL-2R, CD25), the concentration of which, when monitored, can warn of an exacerbation in the course of SLE.[32] Soluble receptors for TNF-α, apart from sIL-2R, neopterins, and intracellular adhesive molecule 1 (IL AM-1), are considered the best markers of immunologic activity.[17]

Determination of the receptors investigated even has a certain advantage over TNF-α concentration measurements, considering that this cytokine is quickly eliminated from the circulation and can be thus difficult or impossible to determine by some methods, especially with bioassays. However, in our study we used the ELISA method by which it is possible to determine the fraction of TNF-α that is associated with soluble receptors.[33] The biological and pathophysiological role of soluble receptors for cytokines is as yet little known. If they are present in serum at a high concentration, they can inhibit the activity of cytokines.[9] Such effect is observed, for instance, for TNF soluble receptors. On the other hand, however, these receptors can increase TNF-α activity by stabilizing its trimetric structure and preventing its dissociation into inactive monomers.[9,17]

It is not fully explained yet if the increase in sTNF-α-R concentration, observed in our SLE patients, is sufficient to alter the biological activity of TNF-α. In individuals exposed to the endotoxin activity, such effect can be then achieved at the sTNF-α-R concentration of 5 ng/ml, which value is considerably higher than in our patients.[34] At lower concentrations, sTNF-α-Rs function as carriers for their ligand and protect it from proteolysis, constituting a reservoir of active or potentially active cytokine.[35] It has been demonstrated that both TNF-α and its production stimulating factors cause at the same time a split-off of soluble receptors,[17] an evidence of which can be the positive correlation between TNF-α and sTNF-Rs observed in our patients. Therefore, the concentration of these receptors in serum is regarded as an indicator of the activity of the whole TNF-α system, and when increased in the result of the system stimulation, it persists longer then high TNF-α concentration.[17]

Our studies indicate that determination of sIL-6R concentration, as opposed to sTNF-α-Rs, is of less importance in SLE. The concentration of the former receptor did not significantly differ in SLE patients and in healthy controls. We have demonstrated, though, a positive correlation between the concentration of sIL-6R and of IL-6, as well as between sIL-6R concentration and disease activity. The results obtained are difficult to interpret since in pathological conditions characterized by high IL-6R concentration high sIL-6R concentration was also observed.[9,36] The pathophysiological role of sIL-6R has not yet been fully determined. Nevertheless, IL-6 is known to bind in physiological conditions with the chain of this receptor, and the complex formed binds them with the endothelial chain $\beta$ (gp130) of the cellular receptor, and only then transduction of the signal in the cell occurs.[37] In *in vitro* studies on myeloma cell lines it has been demonstrated that sensitivity of these cells on IL-5 activity in the presence of sIL-6R increases ten-fold.[38]

We have shown that the concentration of TNF-α, IL-6 and sTNF-α-Rp55 was higher in SLE patients than in healthy individuals, and that these peptides correlate positively with the activity of the disease. Although sIL-6R concentration in SLE patients did not significantly differ, the level of this receptor indicated a positive correlation with the disease activity and IL-6 concentrations.

### References

1. Swaak AJG, Nossent JC, Smeenk RJT. Systemic lupus erythematosus. *Int J Clin Res* 1992; **22**: 190–195.
2. Horwitz DA, Jacob CO. The cytokine network in the pathogenesis of systemic lupus erythematosus and possible therapeutic implications. *Springer Semin Immunopathol* 1994; **16**: 181–200.
3. Linker-Israeli M. Cytokine abnormalities in human lupus. *Clin Immunol Immunopathol* 1992; **63**: 10–12.
4. Handwerger BS, Rus V, da Silva L, Via CS. The role of cytokines in the immunopathogenesis of lupus. *Springer Semin Immunopathol* 1994; **16**: 153–180.
5. Gordon C, Emery P. Cytokines and acute phase response in SLE. *Lupus* 1993; **2**: 345–347.
6. Linker-Israeli M. Cytokine abnormalities in human lupus. *Clin Immunol Immunopathol* 1992; **63**: 10–12.
7. Bazzoni F, Beutler B. The tumor necrosis factor ligand and receptor families. *N Engl J Med* 1996; **334**: 1717–1725.
8. Kishimoto T, Taga T, Akira S. Catokine signal transduction. *Cell* 1994; **76**: 253–262.
9. Heaney ML, Golde DW. Soluble cytokine receptors. *Blood* 1996; **87**: 847–857.
10. Maury CPJ, Teppo A-M. Tumor necrosis factor in the serum of patients with systemic lupus erythematosus. *Arthritis Rheum* 1989; **32**: 146–150.
11. Meijer C, Huysen U, Smeenk RTJ, Swaak AJG. Profiles of cytokines (TNF-α and IL-6) and acute phase proteins (CRP and α 1 AG) related to the disease course in patients with systemic lupus erythematosus. *Lupus* 1993; **2**: 359–365.
12. Al-Janadi M, Al-Balla S, Al Dalaan A, Raziuddin S. Cytokine profile in systemic lupus erythematosus, rheumatoid arthritis and other rheumatic diseases. *J Clin Immunol* 1993; **13**: 58–67.
13. Linker-Israeli M, Deans RJ, Wallace DJ, Prhn J, Oxzeri-Chen T, Klinberg JR. Elevated levels of endogenous IL-6 in systemic lupus erythematosus. *J Immunol* 1991; **147**: 117–123.
14. Spronk PE, ter Borg EJ, Limburg PC, Kallenberg CGM. Plasma concentration of IL-6 in systemic lupus erythematosus: an indicator of disease activity. *Clin Exp Immunol* 1992; **90**: 106–110.
15. Gabay C, Roux-Lombard P, de Moerloose P, Dayer JD, Vischer T, Guerne PA. Absence of correlation between interleukin 6 and C-reactive protein blood levels in systemic lupus erythematosus compared with rheumatoid arthritis. *J Rheumatol* 1993; **20**: 815–821.
16. Metsariune KP, Nordstrom CDE, Konttinen YT, Teppo AM, Fyhrquist FY. Plasma interleukin-6 and renin substrate in reactive arthritis, rheumatoid arthritis and systemic lupus erythematosus. *Rheumatol Int* 1992; **12**: 93–96.
17. Ruiz AD, Tilz GP, Zangerle R, Baier-Bitterlich G, Wachter H, Fuchs D. Soluble receptors for tumour necrosis factor in clinical laboratory diagnosis. *Eur J Haematol* 1995; **54**: 1–8.
18. Aderka D, Engelmann H, Hornik V, Skornick Y, Levo Y, Vallain D, Kustai Y. Increased serum levels of soluble receptors for tumor necrosis factor in cancer patients. *Cancer Res* 1991; **51**: 5602–5607.
19. Samsonov MY, Tilz GP, Egorova O, Reitnegger G, Balabanova RM, Vassomov EL, Nassonova VA, Wachter H, Fuchs D. Serum soluble markers of immune activation and disease activity in systemic lupus erythematosus. *Lupus* 1996; **4**: 29–32.
20. Aderka D, Wysenbeck A, Engellman H, Cope AP, Brennan FM, Molad FY, Hornick V, Levo Y, Maini RN, Feldmann M, Wallach D. Correlation between serum levels of soluble tumor necrosis factor receptor and disease activity in systemic lupus erythematosus. *Arthritis Rheum* 1993; **36**: 1111–1120.
21. Heilig B, Fiehn C, Brockhaus M, Gallati M, Pezzutto A, Hunstein W. Evaluation of soluble tumor necrosis factor (TNF) receptors and TNF receptor antibodies in patients with systemic lupus erythematodes, progressive systemic sclerosis, and mixed connective tissue disease. *J Clin Immunol* 1993; **13**: 321–328.
22. Yasuhawa K, Futatsugi K, Saito T, Valvata H, Narazolin M, Suzuki H, Taga T, Hishimoto T. Association of recombinant soluble IL-6-signal transducer, gp 130, with a complex of IL-6 and soluble IL-6 receptor and establishment of an ELISA for soluble gp 130. *Immunol Lett* 1992; **31**: 123–130.
23. Müllberg J, Schooltink H, Stoyan T, Gunther M, Greave L, Buse G, Machiewicz A, Heinrich PC, Rose-John S. The human soluble interleukin-6 receptor is generated by shedding. *Eur J Immunol* 1993; **23**: 473.
24. Müllberg J, Oberthür W, Lottspeich F, Mehl T, Dittrich T, Graeve L, Heinrich PL, Rose-John S. The soluble human IL-6 receptor. Mutational characterization of the proteolytic cleavage site. *J Immunol* 1934; **152**: 4958–4968.
25. Tan EM, Cohen AS, Fries JF, Masi AT, McShane DJ, Rothfield NF, Schaller JG, Talal N, Winchester RJ. The 1982 revised criteria for the classification of systemic lupus erythematosus. *Arthritis Rheum* 1982; **25**: 1271–1277.
26. Liang MH, Socher SA, Larson MG, Schur PH. Reliability and validity of six systems for the clinical assessment of disease activity in systemic lupus erythematosus. *Arthr Rheum* 1989; **32**: 1107–1118.
27. Malave J, Searles RP, Montano J, Williams RC Jr. Production of tumor necrosis factor/cachectin by peripheral blood mononuclear cells in patients with systemic lupus erythematosus. *Int Arch Allergy Appl Immunol* 1989; **89**: 355–361.
28. Maini RN, Elliott MJ, Charles PJ, Feldmann H. Immunological interventions reveals reciprocal roles for tumor necrosis factor-α and interleukin-10 in rheumatoid arthritis and systemic lupus erythematosus. *Springer Semin Immunol* 1994; **16**: 327–336.
29. Kitani AM, Hara M, Hirose M, Harigami M, Suzuki K, Kawakami M, Kawaguchi Y, Hidaka T, Kawagoe M, Nakamuri T. Autostimulatory effects of IL-6 on excessive B cell differentiation in patients with systemic lupus erythematosus; analysis of IL-6R expression. *Clin Exp Immunol* 1992; 75–83.
30. Nagafuchi H, Suzuki N, Mizushima Y, Sakane T. Constitutive expression of IL-6 receptors and their role in the excessive B cell function in patients with systemic lupus erythematosus. *J Immunol* 1993; **151**: 6525–6534.
31. Tracey JK. TNF and Mae West or: death from too much of a good thing. *N Engl J Med* 1995; **345**: 75–76.
32. Ter Borg EJ, Horst G, Limburg PC, Kallenberg CGM. Changes in plasma levels of interleukin 2 receptors in relation to disease exacerbations and levels of anti-ds DNA and complement in systemic lupus erythematosus. *Clin Exp Immunol* 1990; **82**: 21–26.
33. Engelberts J, Moeller A, Schoen GJM, van der Linde CJ, Bunrman WA. Evaluation of measurement of human TNF in plasma by ELISA. *Lymphokine Cytokine Res* 1991; **10**: 69–76.
34. Van Zee KJ, Kohno T, Fischer, Rock CS, Moldawer LL, Lowry SF. Tumor necrosis factor soluble receptors circulate during experimental and clinical inflammation and can protect against excessive tumor necrosis factor alpha *in vitro* and *in vivo*. *Proc Natl Acad Sci USA* 1992; **89**: 4845.
35. Aderka D, Engellmann H, Maor Y, Brakebusch C, Wallach D. Stabilization of the bioactivity of tumor necrosis factor by its soluble receptors. *J Exp Med* 1992; **175**: 323–239.
36. Honda M. Yamamoto S, Cheng M, Yasukawa K, Suzuki H, Saito T, Osugi Y, Tokunaga T, Hishimoto T. Human soluble IL-6 receptor: its detection and enhanced released by HIV infection. *J Immunol* 1992; **146**: 2175–2189.
37. Kishimoto T, Taga T, Akira S. Catokine signal transduction. *Cell* 1994; **76**: 253–262.
38. Gailard JP, Bataille R, Brailly H, Zuber C, Yasukawa K, Attal M, Marwo N, Taga T, Kishimoto T, Klein B. Increased and highly stable levels of functional soluble interleukin-6 receptor in sera of patients with monoclonal gammapathy. *Eur J Immunol* 1993; **23**: 820–824.

ACKNOWLEDGEMENTS. The authors are grateful to Ms Jolanta Fryczak for excellent technical assistance and to Ms Jolanta Kuczyńska for typing the manuscript.

**Received 30 September 1996; accepted 14 October 1996**
